# Synthesis and optoelectronic properties of benzoquinone-based donor–acceptor compounds

**DOI:** 10.3762/bjoc.15.285

**Published:** 2019-12-04

**Authors:** Daniel R Sutherland, Nidhi Sharma, Georgina M Rosair, Ifor D W Samuel, Ai-Lan Lee, Eli Zysman-Colman

**Affiliations:** 1 Institute of Chemical Sciences, School of Engineering and Physical Sciences, Heriot-Watt University, Edinburgh, EH14 4AS, UK; 2 Organic Semiconductor Centre, EaStCHEM School of Chemistry, University of St Andrews, St Andrews, KY16 9ST, UK; 3 Organic Semiconductor Centre, SUPA School of Physics and Astronomy, University of St Andrews, St Andrews, KY16 9SS, UK

**Keywords:** materials chemistry, physical organic chemistry, spectroscopy, thermally activated delayed fluorescence

## Abstract

Herein, we report a mild and efficient palladium-catalyzed C–H functionalization method to synthesize a series of benzoquinone (BQ)-based charge-transfer (CT) derivatives in good yields. The optoelectronic properties of these compounds were explored both theoretically and experimentally and correlations to their structures were identified as a function of the nature and position of the donor group (*meta* and *para*) attached to the benzoquinone acceptor. Compound **3**, where benzoquinone is *para*-conjugated to the diphenylamine donor group, exhibited thermally activated delayed fluorescence (TADF) with a biexponential lifetime characterized by a prompt ns component and a delayed component of 353 μs.

## Introduction

**Scheme 1:**
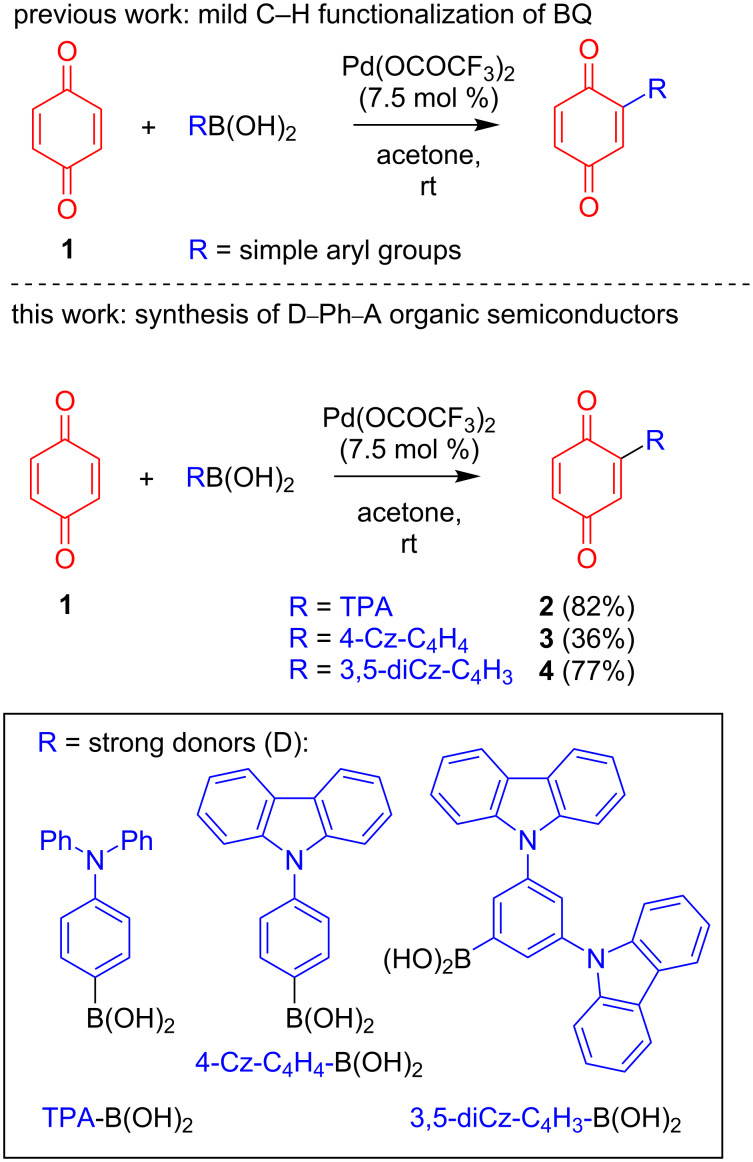
Mild and direct C–H monofunctionalization of BQ **1**: previous [14] and this work.

Substituted benzoquinones and derivatives [1] have generated great interest, in particular due to their redox-active nature and their importance in biological mechanisms [2]. Compounds bearing benzoquinones have found utility in a variety of different fields, such as medicinal chemistry [3], natural products [4], dyes [5], ligands [6–9], oxidation chemistry [10], functional materials [11], and molecular electronics [12–13]. Due to their myriad applications, one of our groups recently developed a controlled and selective direct Pd-catalyzed C–H monofunctionalization of benzoquinone **1** in order to expedite the synthesis of benzoquinone-containing targets (Scheme 1) [2,14]. This direct method not only allows for monofunctionalization of **1** under mild and base-free conditions at ambient temperature, it is also carried out in benign acetone as solvent and does not require any additional oxidant. It circumvents the additional prefunctionalization of the BQ step usually required for Pd cross-couplings [15], while also showing greater functional group tolerance than radical-based methods [16–21].

Nevertheless, only C–H functionalizations with relatively simple aryl, heteroaryl, and cycloalkyl compounds were investigated in our original communication [14]. We therefore aimed to showcase the utility of this mild and direct method by applying it to more challenging systems. With this in mind, we targeted donor (D)–phenylene–acceptor (A) systems that would be of potential interest to the organic semiconductor community. These compounds incorporate strong donors in the form of triphenylamine (TPA) and carbazole (Cz), as can be seen in Scheme 1. Since the corresponding arylboronic acids are readily accessible, we envisaged that our Pd-catalyzed direct C–H functionalization method would be ideal for attempting a facile and direct route to a series of novel substituted benzoquinone-based charge-transfer derivatives, with the aim of exploring their electroluminescent (EL) properties.

In EL devices, such as organic light-emitting diodes (OLEDs), there are three main exciton-harvesting mechanisms for organic compounds that offer the potential to expand beyond the 25% internal quantum efficiency (IQE) offered by fluorophores: (1) phosphorescence, frequently mediated by a rare noble metal complex, such as those based on iridium(III) [22]; (2) triplet–triplet annihilation (TTA) [23], or (3) TADF. The maximum IQE for TTA is 62.5%, whereas for TADF, it is as high as 100% [24]. TADF emitter development has thus emerged as an effective avenue to achieve high performance in OLEDs. This is possible as these compounds possess very small exchange energies between their excited singlet and triplet states (Δ*E*_ST_), which facilitates reverse intersystem crossing (RISC), in which singlet excitons are generated from triplet excitons. In order to obtain a small Δ*E*_ST_, the molecular design of the TADF emitter requires that the highest occupied molecular orbital (HOMO) must be spatially well-separated from the lowest unoccupied molecular orbital (LUMO), such that the exchange integral is minimized [25–26]. Due to a ′trade-off′ between Δ*E*_ST_ and high fluorescence radiative rates [27], it is difficult to realize highly efficient TADF emitters, which is especially the case for red emitters where nonradiative rates are high due to the well-known energy gap law [28].

**Figure 1:**
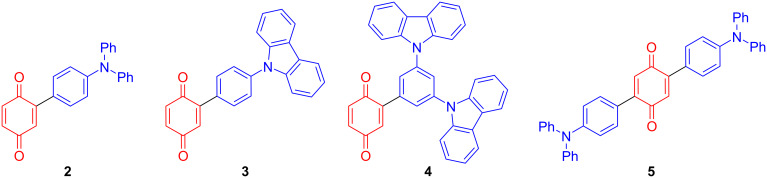
Benzoquinone derivatives synthesized for this study, with the donor in red and the benzoquinone acceptor in blue.

While anthraquinone-based charge-transfer compounds have recently been shown to exhibit TADF that then translated to their use in highly efficient, orange-to-red OLEDs (λ_EL_ ranging from 584–637 nm, EQE_max_ ranging from 6.9–12.5%) [29], to the best of our knowledge, no benzoquinone-based compounds have been investigated in the context of TADF emitter design. The stronger acceptor character of benzoquinone is attractive because it should lead to a more red-shifted emission. As described above, with a mild and efficient method to functionalize BQs now in hand, we decided to exploit the C–H functionalization methodology developed within our group [14] to readily synthesize a series of benzoquinone-based charge-transfer compounds (Figure 1), with the aim of exploring their optoelectronic properties.

## Results and Discussion

### Synthesis

**Scheme 2:**
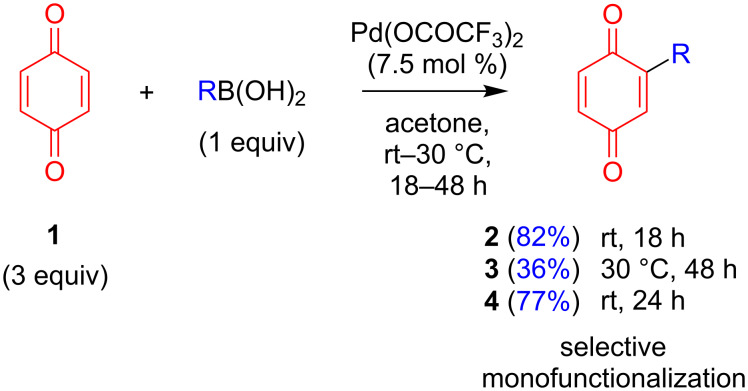
Synthesis of **2**–**4** via mild and direct C–H monofunctionalization of BQ (**1**).

**Scheme 3:**
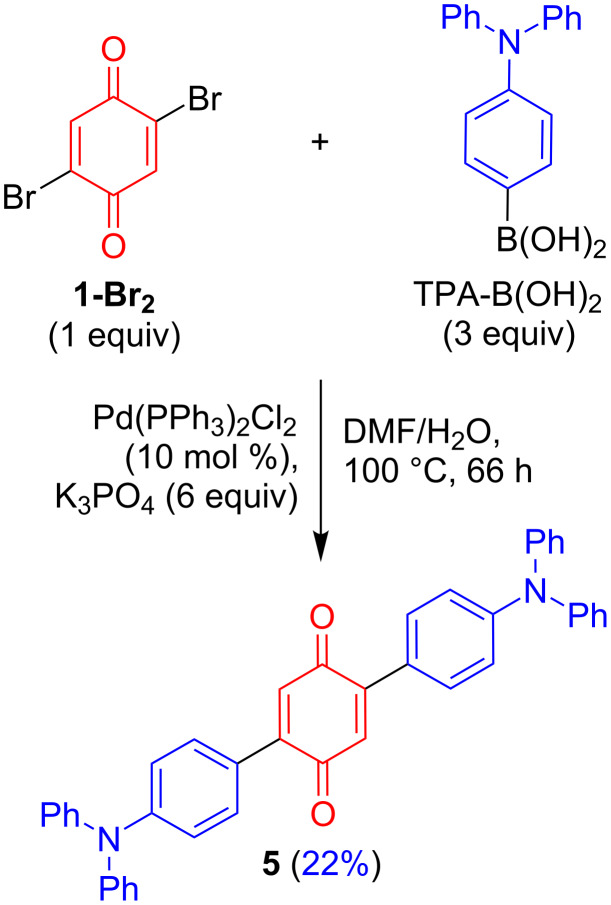
Synthesis of **5** via double Suzuki coupling.

**Figure 2:**
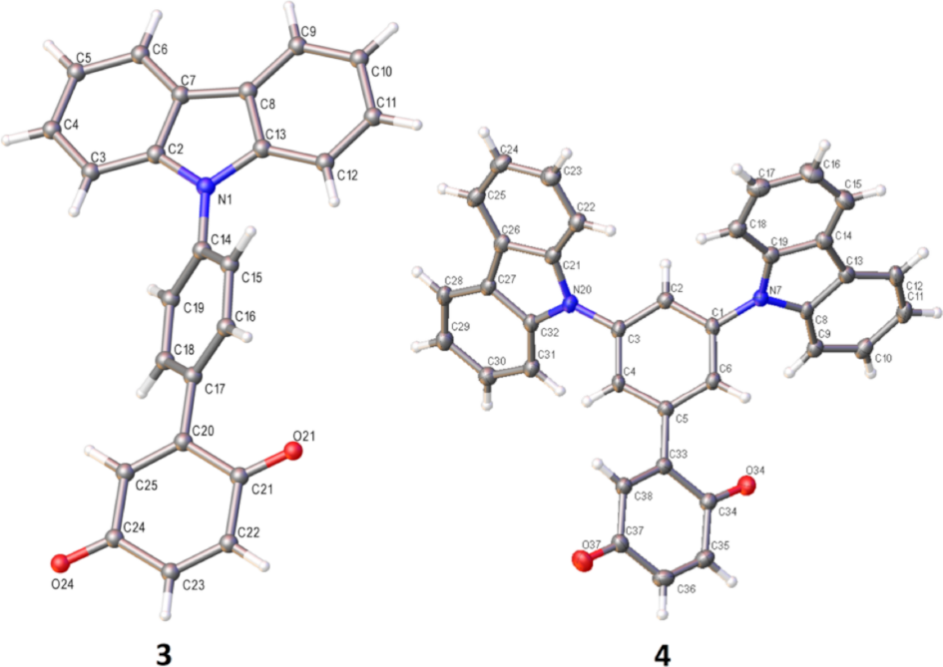
Crystal structures of **3** and **4**.

We initiated our studies by synthesizing monofunctionalized donor–acceptor compounds **2**–**4**, using the Pd(II)-catalyzed direct C–H functionalization method described below (Scheme 2). To our delight, compounds **2** and **4** were readily obtained in high yields and in one step from **1** (82% for **2** and 77% for **4**) using this mild method. Compound **3** was also successfully formed from **1** in 36% yield, although a slightly higher temperature of 30 °C and longer reaction time (48 h) were required. In order to compare the monofunctionalized BQ **2** with a difunctionalized analogue, **5** was also prepared for our investigation. As 2,5-difunctionalized BQs with electron-donating groups are not accessible via the Pd(II)-catalyzed direct C–H functionalization method utilized in Scheme 2 (since the selectivity for difunctionalization is dependent on the electronics of the substituent added to BQ) [14], **5** was instead furnished from the dibromo-functionalized BQ **1-Br_2_** using a double Suzuki coupling. As expected, much harsher conditions (100 °C) were required to achieve conversions under Pd(0) catalysis to produce **5** in 22% yield (Scheme 3). Compounds **2**–**5** are novel and designed to contain a strong acceptor (BQ, in red) and a strong donor group (R, in blue): TPA in **2** and **5**, 4-Cz-C_4_H_4_ in **3**, and 3,5-diCz-C_4_H_3_ in **4**. Crystal structures were successfully obtained for compounds **3** and **4**, which show the expected twisting of the donor carbazoles with respect to the bridging benzene of ca. 44° in both **3** and **4** (Figure 2).

### Theoretical properties

**Figure 3:**
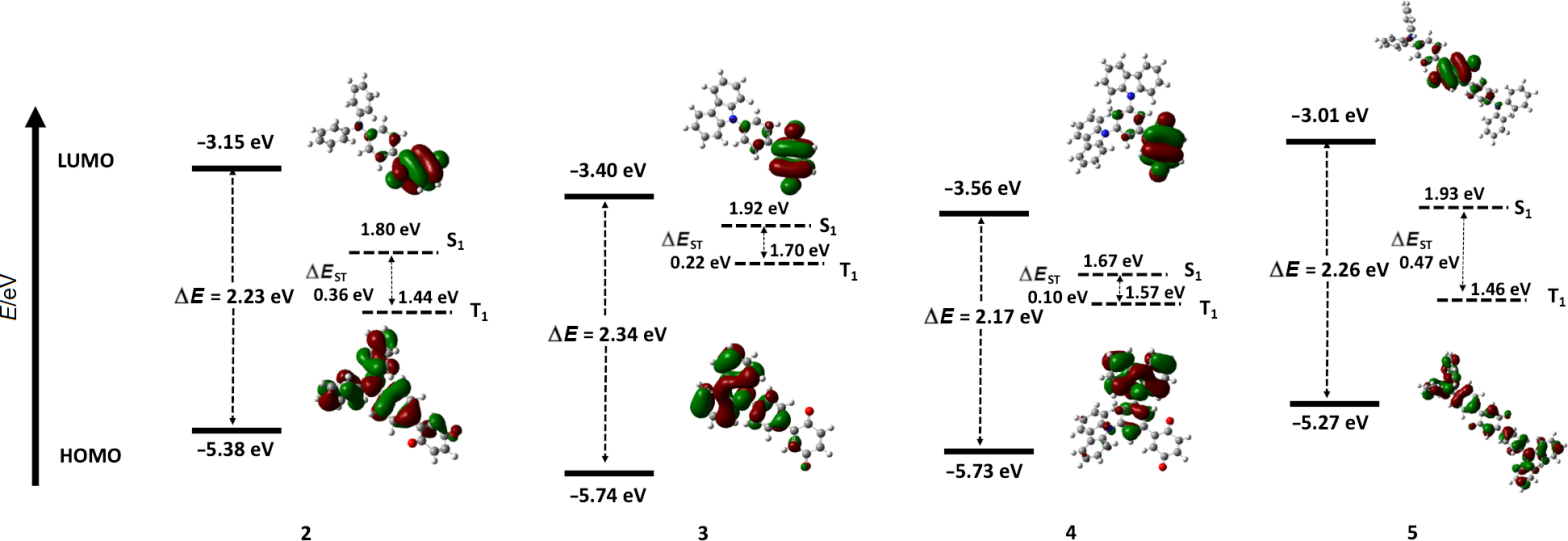
HOMO/LUMO and S_1_/T_1_ energies as well as HOMO/LUMO electron density distribution profiles of **2**–**5**.

Density functional theory (DFT) calculations were performed in the gas phase to assess the electronic structures of **2**–**5** (see Supporting Information File 1 for details). The S_1_ and T_1_ excited states were calculated from the optimized ground-state structure using the Tamm Dancoff approximation (TDA) to time-dependent density functional theory (TD-DFT) [30–31]. In all derivatives, the LUMO is mainly localized on the strongly electron-accepting BQ moiety (Figure 3). The HOMO is mainly located on the donor moieties. The HOMO is more delocalized in **5** and **2** compared to **3** and **4**, a function of the more planar conformation of the TPA units despite the stronger donor strength compared to Cz. Time-dependent calculations using the TDA approach predicted Δ*E*_ST_ values of 0.36, 0.22, 0.10, and 0.47 eV for **2**, **3**, **4** and **5**, respectively. The small Δ*E*_ST_ values for **3** and **4** could facilitate efficient ISC/RISC, mediated by a TADF mechanism. The relatively large Δ*E*_ST_ values for **2** and **5** were the result of an increased conjugation of the diarylamine donors with the BQ acceptor in these compounds, leading to the enhanced overlap of the HOMO and LUMO wave functions.

### Electrochemical properties

**Figure 4:**
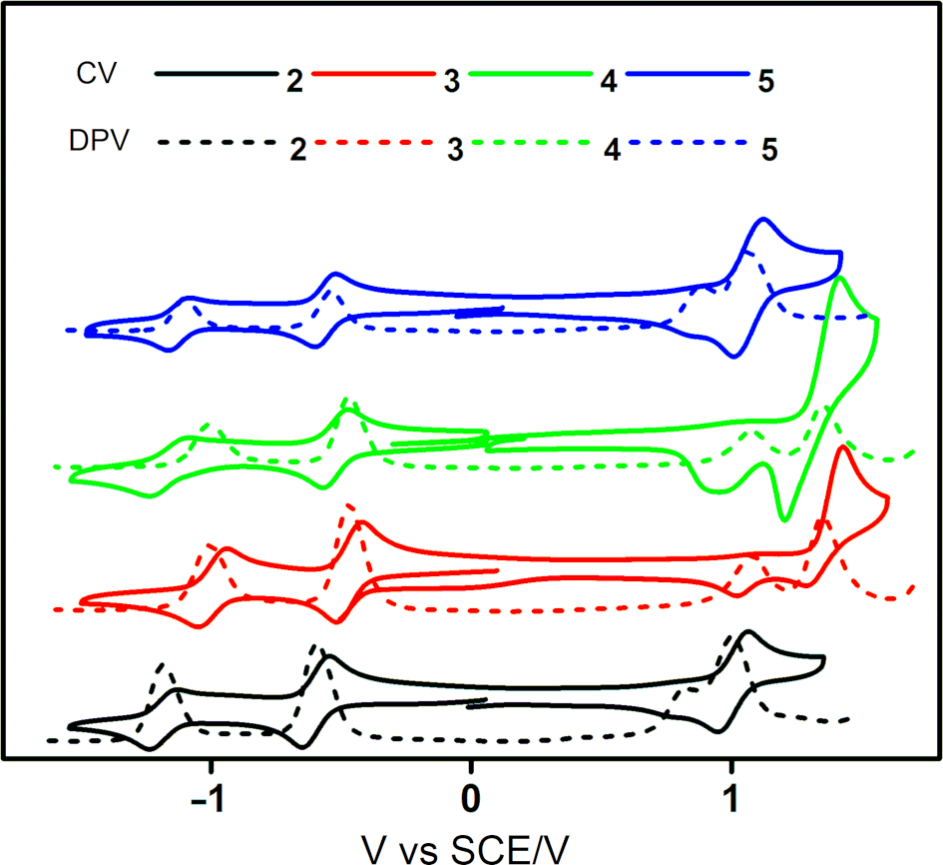
Cyclic voltammograms and differential pulse voltammograms of **2**–**5** in degassed DCM (scan rate = 100 mV·s^−1^).

The electrochemical behavior of **2**–**5** was studied by cyclic voltammetry and differential pulse voltammetry in degassed DCM with tetrabutylammonium hexafluorophosphate as the supporting electrolyte. The cyclic voltammograms (CVs) and differential pulse voltammograms (DPVs) are shown in Figure 4 and the data are summarized in Table 1. Upon scanning to negative voltage, all four compounds exhibited two highly reversible reduction waves, a typical behavior of the BQ moiety in aprotic solvents [32]. Based on DFT calculations and a comparison with the literature data [32–33], the first reduction wave was assigned to the formation of the hydroquinone anion at *E*_red_^1^ from −0.47 V for **4** to −0.59 V for **2**, while the second reduction wave, observed at *E*_red_^2^ from −1.00 V for **3** to −1.18 V for **2**, was assigned to the formation of the hydroquinone dianion. LUMO energies of the four compounds are thus in the order of −4.20 eV for compounds **2** and **5** and −4.30 eV for compounds **3** and **4** (Table 1). At positive potentials, the two diphenylamine derivatives (**2** and **5**) showed reversible one-electron oxidation waves, while the two carbazole donor derivatives (**3** and **4**) showed irreversible oxidation waves, which is a function of the electrochemically unstable carbazole-based radical cation that can subsequently undergo dimerization [34]. The oxidation waves shifted cathodically upon increasing the donor strength from carbazole (**3** and **4**) to diphenylamine (**2** and **5**). Similarly, the presence of two donors in **4** and **5** resulted in a cathodic shift of the oxidation waves compared to **2** and **3**, respectively. The electrochemical gaps Δ*E*_redox_ of the four derivatives are thus 1.61, 1.83, 1.85, and 1.58 V for **2**, **3**, **4**, and **5**, respectively. The observed HOMO and LUMO energies of **2**–**5** are in good agreement with the calculated values (Figure 3).

**Table 1:** Electrochemical properties of **2**–**5**.

Compound	*E*_ox_^a^/V	*E*_red_^1a^/V	*E*_red_^2a^/V	E_HOMO_^b^/eV	E_LUMO_^b^/eV	Δ*E*_redox_^c^/eV

**2**	1.02	−0.59	−1.18	−5.82	−4.21	1.61
**3**	1.35	−0.48	−1.00	−6.15	−4.32	1.83
**4**	1.38	−0.47	−1.01	−6.18	−4.33	1.85
**5**	1.05	−0.53	−1.09	−5.85	−4.27	1.58

^a^Oxidation and reduction peak potentials from DPV in DCM with 0.1 M [*n-*Bu_4_N]PF_6_ as supporting electrolyte and Fc/Fc^+^ as internal reference (0.46 V vs SCE) [35]. ^b^The HOMO and LUMO energies were determined using the relation *E*_HOMO_/*E*_LUMO_ = −(*E*_ox_/*E*_red_^1^ + 4.8 eV) [36]. ^c^Δ*E*_redox_ = |*E*_HOMO_ − E_LUMO_|.

### Photophysical properties

**Figure 5:**
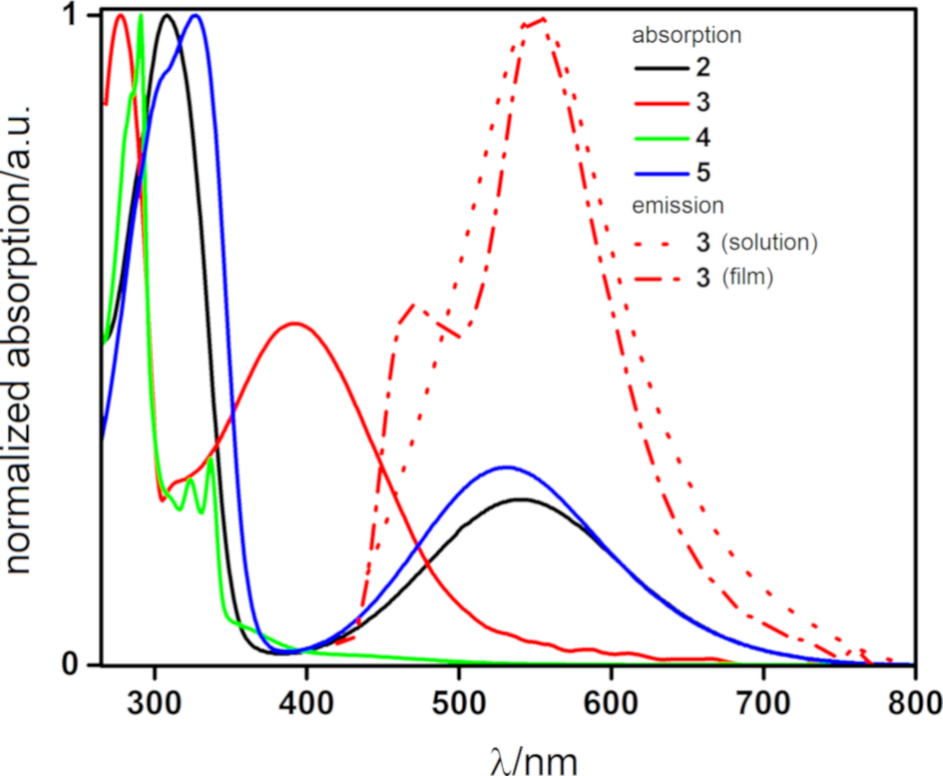
UV–vis absorption spectra of **2–5** in DCM and photoluminescence spectrum of **3** in degassed DCM and in 10 wt %-doped films in a PMMA matrix (λ_exc_ = 400 nm).

We next investigated the photophysical properties of **2**–**5**. The UV–vis absorption and photoluminescence (PL) spectra in degassed DCM solution are shown in Figure 5, and the data are summarized in Table 2. In the absorption spectra, **2** and **5** both showed intense π–π* absorption bands localized on the benzophenone at 326 and 310 nm, respectively [37]. The extra TPA donor in **5** contributed to a red-shifting of this absorption band. There were low-energy CT bands from the TPA donor to the BQ acceptor at 530 and 541 nm, respectively, for **5** and **2**. A similar relationship existed for **3** and **4**. The π–π* absorption bands of these two compounds are blue-shifted compared to their TPA congeners at 280 and 291 nm for **3** and **4**, respectively. There are likewise low-energy CT bands at 398 nm for **3** that are blue-shifted compared to those found for **2** and **5**. Charge-transfer was expected to be weaker in compound **4**, where the donor groups were *meta* disposed with respect to the acceptor. Indeed, there was only a very poorly absorptive CT band at 370 nm for this compound, which reflected the poor conjugation between the donor and the acceptor.

**Table 2:** Optoelectronic properties of **2**–**5**.

Compound	λ_abs_^a^/nm	λ_PL_^b^/nm	Φ_PL_^c^/%	λ_PL_^d^/nm	Φ_PL_^d^/%	τ_p_; τ_d_^d^/ns; μs

**2**	310, 541	–			–	–
**3**	280, 398	550	4	450, 550	6	19.4; 352.6
**4**	291, 370	–			–	–
**5**	326, 530	–			–	–

^a^In DCM at 298 K. ^b^In degassed DCM at 298 K. ^c^[Ru(bpy)_3_]Cl_2_ (aq) was used as reference (Φ_PL_ = 4%) [38]. Values quoted are in degassed solutions, which were prepared by three freeze-pump-thaw cycles. ^d^Thin films were prepared by spin coating 10 wt %-doped samples in PMMA. Φ_PL_ values were determined using an integrating sphere, λ_exc_ = 400 nm.

Compounds **2**, **4**, and **5** were very poorly luminescent and so their emission could not be quantified. Compound **3** exhibited a broad and unstructured emission in a degassed DCM solution (λ_PL_ = 550 nm), characteristic of an excited state with significant CT character. The photoluminescence quantum yield (Φ_PL_) of **3** is poor at 4%.

We next investigated the solid-state PL behavior of **3** in a 10 wt %-doped thin film using PMMA as host matrix. The PL spectrum of **3** exhibited two bands, one at 550 nm, corresponding to the emission of **3** and a second high-energy band at 450 nm that was attributed to the PMMA host matrix [39]. The narrower emission profile of **3** in the solid state than in DCM solution could be due to the suppression of various nonradiative vibrational modes in the inert host matrix PMMA.

**Figure 6:**
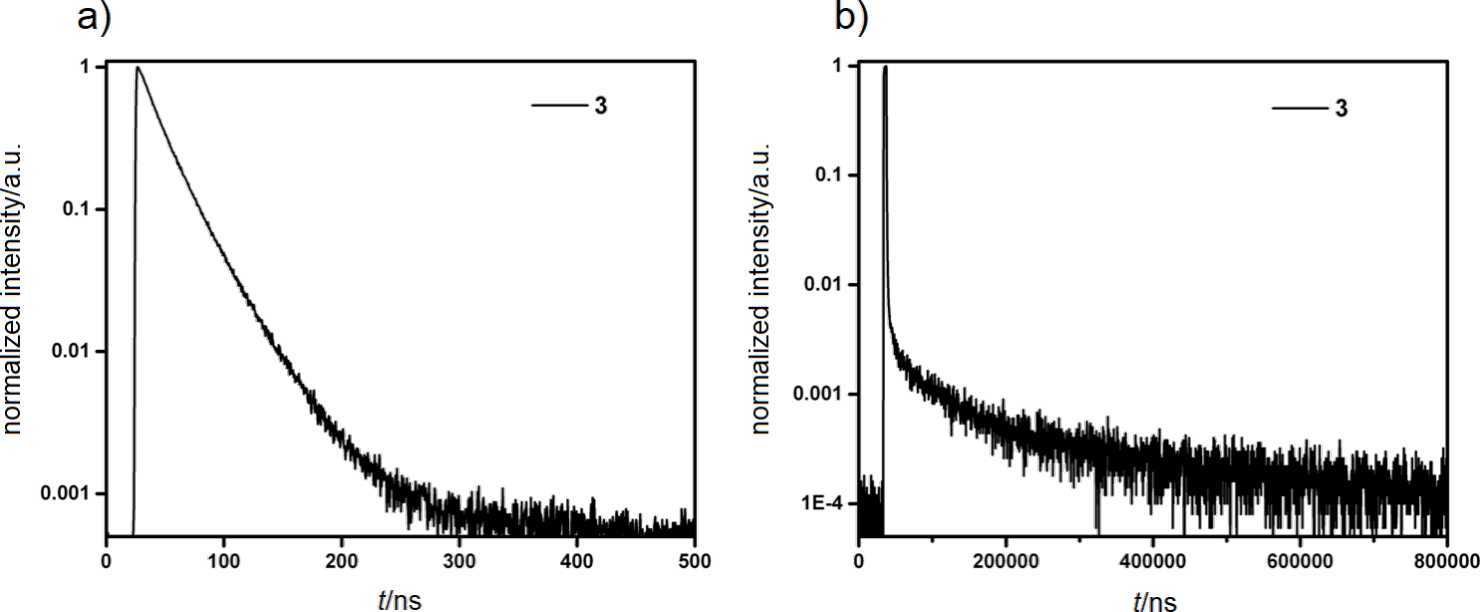
Time-resolved PL plots. a) Prompt decay and b) delayed decay curve of **3** in thin film (λ_exc_ = 378 nm).

Compound **3** showed a low Φ_PL_ of 6% under nitrogen atmosphere. Time-resolved PL measurements using a combination of TCSPC and MCS techniques (at λ_PL_ = 550 nm) revealed two emission decay lifetimes: a prompt component, τ_p_, of 19.4 ns and a very long delayed component, τ_d_, of 352 μs. The two timescales of luminescence are a hallmark of delayed fluorescence behavior (Figure 6).

The PL behavior of the four compounds was disappointing, especially compared to the high Φ_PL_ values and deep red emission reported for anthraquinone-based TADF emitters [29]. For the nonemissive complexes, it is possible that this was due to ISC being much faster than RISC. For benzoquinone, ISC occurs within 10 ps [40], with efficiency close to unity [41–42], implying that the nonradiative ISC channel of excitons from S_1_ to T_1_ is always preferred over the radiative decay from the S_1_ state. For compound **3**, we observed a prompt lifetime of 19.4 ns, which indicated there was not ultrafast ISC. This is consistent with **3** having higher Φ_PL_ than the other compounds.

## Conclusion

Three novel monofunctionalized donor–acceptor BQ derivatives **2**–**4** were synthesized using an efficient Pd-catalyzed C–H functionalization method, and novel 2,5-difunctionalized BQ derivative **5** was also obtained via Suzuki coupling. The theoretical, electrochemical, and photophysical properties of the donor–acceptor compounds **2**–**5** were determined in solution and thin film. While compounds **2**, **4**, and **5** were very poorly emissive, **3** showed a green emission with a biexponential lifetime characterized by a prompt nanosecond component and a delayed component of 353 μs, indicative of a TADF emission.

## Experimental

**4'-(Diphenylamino)[1,1'-biphenyl]-2,5-dione (2):** To a 4 mL vial equipped with a magnetic stirring bar were added 4-(diphenylamino)phenylboronic acid (52.0 mg, 0.18 mmol, 1 equiv), benzoquinone (58.4 mg, 0.54 mmol, 3 equiv), and Pd(TFA)_2_ (9.0 mg, 0.027 mmol, 15 mol %). Acetone (2.2 mL) was added and the reaction mixture was stirred for 18 h at rt. The crude mixture was directly adsorbed onto a silica gel column and purified using toluene as the eluent. Further purification using glass-backed silica plates with toluene as the eluent yielded the title product **1** as a dark purple crystalline solid (52 mg, 0.15 mmol, 82%). mp 160–165 °C; IR ν_max_ (cm^−1^): 3035, 1652, 1648, 1579, 1505, 1488; ^1^H NMR (400 MHz, CDCl_3_) δ 7.42–7.37 (m, 2H), 7.33–7.28 (m, 4H), 7.18–7.13 (m, 4H), 7.13–7.08 (m, 2H), 7.07–7.03 (m, 2H), 6.85–6.77 ppm (m, 3H); ^13^C{^1^H}NMR [6–9] (101 MHz, CDCl_3_) δ 187.7 (C), 187.4 (C), 150.2 (C), 147.0 (C), 145.2 (C), 137.2 (CH), 136.5 (CH), 130.53 (CH), 130.52 (CH), 129.7 (CH), 125.7 (CH), 125.3 (C), 124.3 (CH), 121.4 ppm (CH); FTMS (+ p NSI, *m*/*z*): [M + H]^+^ calcd for C_24_H_18_NO_2_, 352.1332; found, 352.1333; HRESIMS-TOF (*m*/*z*): [M + H]^+^ calcd for C_24_H_17_NO_2_, 352.1338; found, 352.1337.

**4'-(9*H*-Carbazol-9-yl)[1,1'-biphenyl]-2,5-dione (3):** To a sealed tube equipped with a magnetic stirring bar were added 4-(9*H*-carbazol-9-yl)phenylboronic acid (172 mg, 0.60 mmol, 1 equiv), benzoquinone (195 mg, 1.80 mmol, 3 equiv), and Pd(TFA)_2_ (29.9 mg, 0.09 mmol, 15 mol %). Acetone (7.2 mL) was added and the reaction mixture stirred for 48 h at 30 °C. The crude mixture was adsorbed directly onto a silica gel column and subsequent purification with DCM/petroleum ether/toluene, 1:1:0.1 to 2:1:0.1, v/v as the eluent yielded the title product **3** as a dark red crystalline solid (75 mg, 0.21 mmol, 36%). mp 184–188 °C; ^1^H NMR (400 MHz, CDCl_3_) δ 8.15 (ddd, *J* = 7.9, 1.2, 0.7 Hz, 2H), 7.78–7.73 (m, 2H), 7.72–7.67 (m, 2H), 7.50 (app dt, *J* = 8.3, 0.9 Hz, 2H), 7.43 (ddd, *J* = 8.3, 7.1, 1.2 Hz, 2H), 7.32 (ddd, *J* = 7.9, 7.1, 1.1 Hz, 2H), 7.00 (d, *J* = 2.4 Hz, 1H), 6.94 (d, *J* = 10.1 Hz, 1H), 6.89 ppm (dd, *J* = 10.1, 2.4 Hz, 1H); ^13^C{^1^H} NMR (101 MHz, CDCl_3_) δ 187.5 (C), 186.7 (C), 145.1 (C), 140.7 (C), 139.9 (C), 137.3 (CH), 136.6 (CH), 132.9 (CH), 131.5 (C), 131.0 (CH), 127.0 (CH), 126.3 (CH), 123.9 (C), 120.58 (CH), 120.57 (CH), 110.0 ppm (CH); HRESIMS-TOF (*m*/*z*): [M + H]^+^ calcd for C_24_H_15_NO_2_, 350.1181; found, 350.1176.

**3',5'-Di(9*H*-carbazol-9-yl)[1,1'-biphenyl]-2,5-dione (4):** To a sealed tube equipped with a magnetic stirring bar were added 3,5-di(9*H*-carbazol-9-yl)phenylboronic acid (123 mg, 0.28 mmol, 1 equiv), benzoquinone (90.8 mg, 0.84 mmol, 3 equiv), and Pd(TFA)_2_ (9.3 mg, 0.028 mmol, 10 mol %). Acetone (3.4 mL) was added and the reaction mixture stirred for 24 h at rt. The crude mixture was adsorbed directly onto a silica gel column and subsequent purification using DCM as the eluent yielded the title product **4** as a dark red crystalline solid (111 mg, 0.22 mmol, 77%). mp 155–160 °C; ^1^H NMR (400 MHz, CDCl_3_) δ 8.17 (d, *J* = 7.8 Hz, 2H), 7.97 (s, 1H), 7.82 (d, *J* = 1.9 Hz, 1H), 7.64 (dd, *J* = 8.4, 0.7 Hz, 2H), 7.51–7.45 (m, 2H), 7.37–7.31 (m, 2H), 7.05 (dd, *J* = 2.3, 1.2 Hz, 1H), 6.96–6.84 ppm (m, 2H); ^13^C{^1^H} NMR (101 MHz, CDCl_3_) δ 187.1 (C), 186.1 (C), 144.4 (C), 140.6 (C), 139.8 (CH), 137.1 (CH), 136.7 (C), 136.0 (CH), 133.6 (CH), 126.5 (CH), 126.3 (CH), 126.2 (CH), 124.0 (C), 120.9 (CH), 120.7 (CH), 109.8 ppm (CH); HRASAP^+^MS-TOF (*m*/*z*): [M + H]^+^ calcd for C_36_H_22_N_2_O_2_, 515.1760; found, 515.1756; HRESIMS-TOF (*m*/*z*): [M + H]^+^ calcd for C_36_H_22_N_2_O_2_, 515.1760; found, 515.1760.

**4,4''-Bis(diphenylamino)[1,1':4',1''-terphenyl]-2',5'-dione (5):** To a Schlenk tube equipped with a magnetic stirring bar were added 2,5-dibromocyclohexa-2,5-diene-1,4-dione (251 mg, 0.94 mmol, 1 equiv), 4-(diphenylamino)phenylboronic acid (809 mg, 2.80 mmol, 3 equiv), K_3_PO_4_ (1.20 g, 5.65 mmol, 6 equiv), and Pd(PPh_3_)_2_Cl_2_ (66.0 mg, 0.09 mmol, 10 mol %). A mixture of water (2.4 mL) and DMF (9.6 mL) was degassed by sparging with argon for 10 min before it was added to the Schlenk tube. The reaction mixture was sparged with argon for a further 5 min before the Schlenk tube was sealed and the reaction mixture stirred for 66 h at 100 °C. The crude mixture was concentrated under reduced pressure, dissolved in toluene (20 mL), and treated with 2,3-dichloro-5,6-dicyano-*p*-benzoquinone (427 mg, 1.88 mmol, 2 equiv) for 30 min at rt with stirring. The reaction mixture was concentrated under reduced pressure and adsorbed directly onto a silica gel column, and subsequent purification with DCM/petroleum ether, 1:2 to 2:1, v/v as the eluent yielded the title product **3** as a dark purple solid (121 mg, 0.20 mmol, 22%). mp 206–210 °C; IR ν_max_ (cm^−1^): 3034, 2924, 1644, 1582, 1504, 1485; ^1^H NMR (400 MHz, CDCl_3_) δ 7.48–7.44 (m, 4H), 7.33–7.28 (m, 8H), 7.18–7.14 (m, 8H), 7.13–7.08 (m, 4H), 7.08–7.04 (m, 4H), 6.88 ppm (s, 2H); ^13^C{^1^H} NMR (101 MHz, CDCl_3_) δ 187.7 (C), 150.0 (C), 147.1 (C), 144.8 (C), 131.1 (CH), 130.6 (CH), 129.65 (CH), 125.69 (CH), 125.4 (C), 124.2 (CH), 121.5 ppm (CH); FTMS (+ p NSI, *m*/*z*): [M + H]^+^ calcd for C_42_H_31_N_2_O_2_, 595.2380; found, 595.2369; HRESIMS-TOF (*m*/*z*): [M + H]^+^ calcd for C_42_H_30_N_2_O_2_, 595.2385; found, 595.2383.

## Supporting Information

The research data supporting this publication can be accessed at https://doi.org/10.17630/178f3a7c-4717-43f0-a145-049675825e1a.

File 1 NMR spectra and supplementary photophysical measurements.

File 2 Crystallographic data for **3** (CCDC 1836680).

File 3 Crystallographic data for **4** (CCDC 1836681).
