# Supplementary material for: Synthesis and optoelectronic properties of benzoquinone-based donor–acceptor compounds
Source: Beilstein J Org Chem. 2019 Dec 4;15:2914–21. doi: 10.3762/bjoc.15.285 (PMC6902851; doi:10.3762/bjoc.15.285)
Supplement: File 1 — NMR spectra and supplementary photophysical measurements. [file Beilstein_J_Org_Chem-15-2914-s001.pdf]

BEILSTEIN JOURNAL OF ORGANIC CHEMISTRY

Supporting Information

for

Synthesis and optoelectronic properties of benzoquinone-based donor–acceptor compounds

Daniel R. Sutherland, Nidhi Sharma, Georgina M. Rosair, Ifor D. W. Samuel, Ai-Lan Lee and Eli Zysman-Colman

Beilstein J. Org. Chem. **2019,** *15,* 2914–2921. doi:10.3762/bjoc.15.285

NMR spectra and supplementary photophysical measurements

License and Terms: This is a supporting information file under the terms of the Creative Commons Attribution License (http://creativecommons.org/licenses/by/4.0). Please note that the reuse, redistribution and reproduction in particular requires that the authors and source are credited.
The license is subject to the *Beilstein Journal of Organic Chemistry* terms and conditions: (https://www.beilstein-journals.org/bjoc)

Table of Contents

1. Crstallographic data S2

2. ^{1}H and ^{13}C NMR spectra...S7

3. Photophysical characterisation..S11

4. DFT modelling...S11

5. References...S22

Crystallographic data

Crystal structure of 4'-(9*H*-carbazol-9-yl)-[1,1'-biphenyl]-2,5-dione (3)

(see Supporting Information File 2)

Single crystals of $C_{24}H_{15}NO_2$ **3** were grown from CH_2Cl_2/hexane. A suitable crystal was selected and mounted on a Bruker APEX-II CCD' diffractometer. The crystal was kept at 100.0 K during data collection. Using Olex2[1], the structure was solved with the XS [2] structure solution program using Intrinsic Phasing and refined with the XL [3] refinement package using Least Squares minimisation.

The dione ring is disordered over 2 positons which additionally lies over a two-fold axis and therefore constrains the occupancies of both conformations to be 50%.

Crystallographic data have been deposited at the Cambridge Crystallographic Data Centre and assigned to the following deposition number: CCDC 1836680

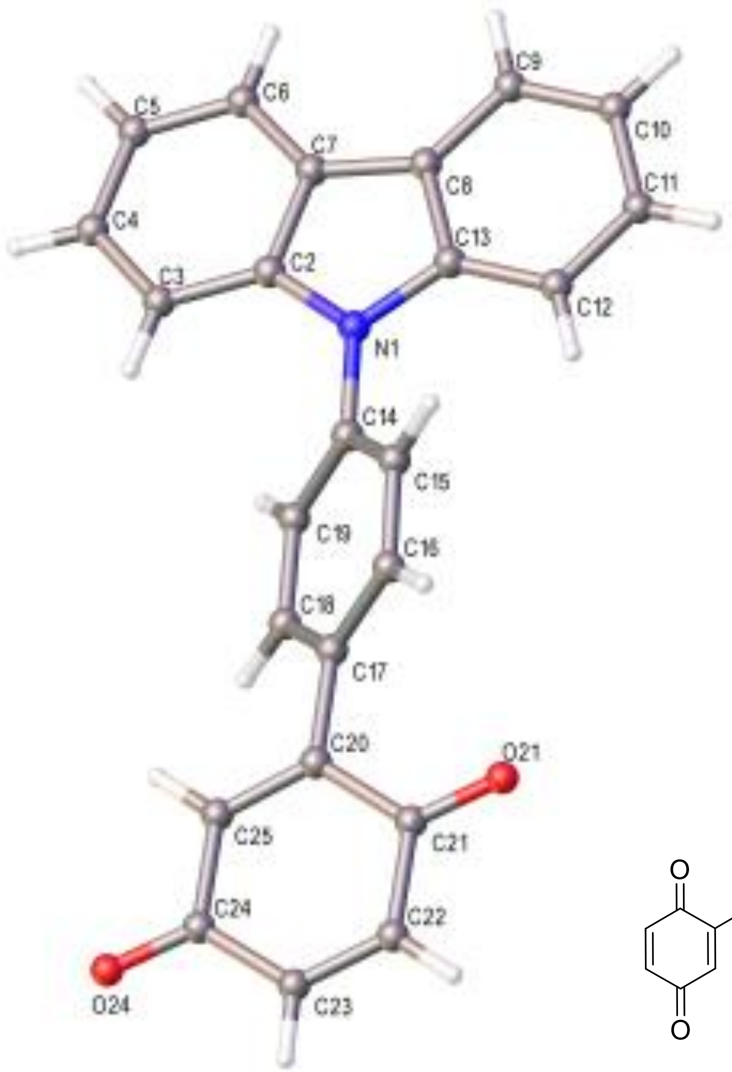

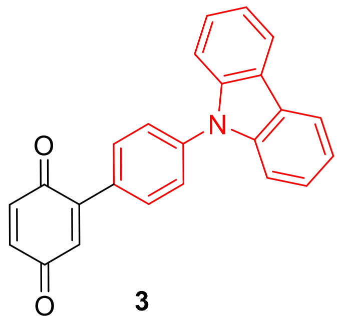

3

Empirical formula	$C_{24}H_{15}NO_2$
Formula weight	349.37
Temperature/K	100
Crystal system	monoclinic
Space group	C2/c
a/Å	9.4955(3)
b/Å	25.1250(9)
c/Å	22.1462(8)
α/°	90
β/°	101.7566(17)
γ/°	90
Volume/Å^3	5172.7(3)
Z	12
ρ_{calc}g/cm^3	1.346
μ/mm^{-1}	0.086
F(000)	2184.0
Crystal size/mm^3	0.34 × 0.32 × 0.08
Radiation	MoKα (λ = 0.71073)
2Θ range for data collection/°	4.672 to 56.006
Index ranges	$-12 \leq h \leq 12$, $-33 \leq k \leq 33$, $-29 \leq l \leq 28$

Reflections collected	45962
Independent reflections	6250 [R_{int} = 0.0418, R_{sigma} = 0.0313]
Data/restraints/parameters	6250/0/397
Goodness-of-fit on F^2	1.025
Final R indexes [I>=2σ (I)]	R_1 = 0.0448, wR_2 = 0.1073
Final R indexes [all data]	R_1 = 0.0691, wR_2 = 0.1196
Largest diff. peak/hole / e Å^{-3}	0.32/-0.28

Crystal structure of 3',5'-di(9H-carbazol-9-yl)-[1,1'-biphenyl]-2,5-dione (4)

(see Supporting Information File 3)

Single crystals of $C_{36}H_{22}N_2O_2$ **4** were grown from ethylacetate/hexane. A suitable crystal was selected and mounted on a SuperNova, Dual source Atlas diffractometer. The crystal was kept at 120.0 K during data collection. Using Olex2 [1], the structure was solved with the XS [2] structure solution program using Intrinsic Phasing and refined with the XL [3] refinement package using Least Squares minimization.

Crystallographic data have been deposited at the Cambridge Crystallographic Data Centre and assigned to the following deposition number: CCDC number: 1836681

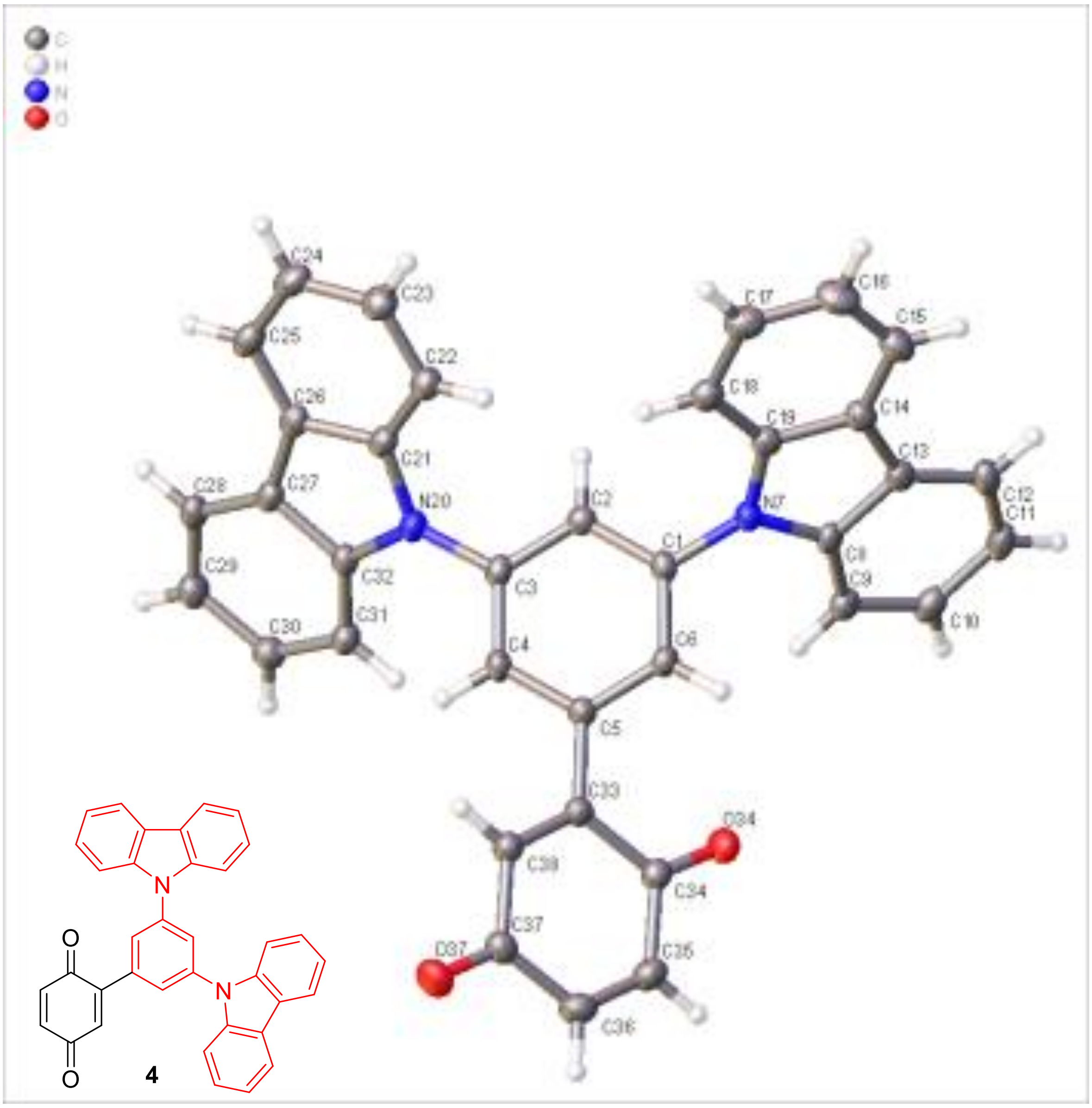

Empirical formula	$C_{36}H_{22}N_2O_2$
Formula weight	514.55
Temperature/K	120.00(10)
Crystal system	triclinic
Space group	P-1
a/Å	8.0600(3)
b/Å	14.4246(6)
c/Å	22.2318(8)
α/°	88.315(3)
β/°	79.652(3)
γ/°	86.713(3)
Volume/Å^3	2538.01(17)
Z	4
ρ_{calc}g/cm^3	1.347
μ/mm^{-1}	0.663
F(000)	1072.0
Crystal size/mm^3	0.491 × 0.073 × 0.023

Radiation	CuKα (λ = 1.54184)
2Θ range for data collection/°	6.138 to 152.814
Index ranges	$-10 \le h \le 9$, $-18 \le k \le 17$, $-28 \le l \le 26$
Reflections collected	41235
Independent reflections	10517 [R_{int} = 0.0801, R_{sigma} = 0.0635]
Data/restraints/parameters	10517/0/721
Goodness-of-fit on F^2	1.013
Final R indexes [I>=2σ (I)]	R_1 = 0.0553, wR_2 = 0.1422
Final R indexes [all data]	R_1 = 0.0676, wR_2 = 0.1522
Largest diff. peak/hole / e Å^{-3}	0.26/-0.34

^{1}H and ^{13}C NMR spectra

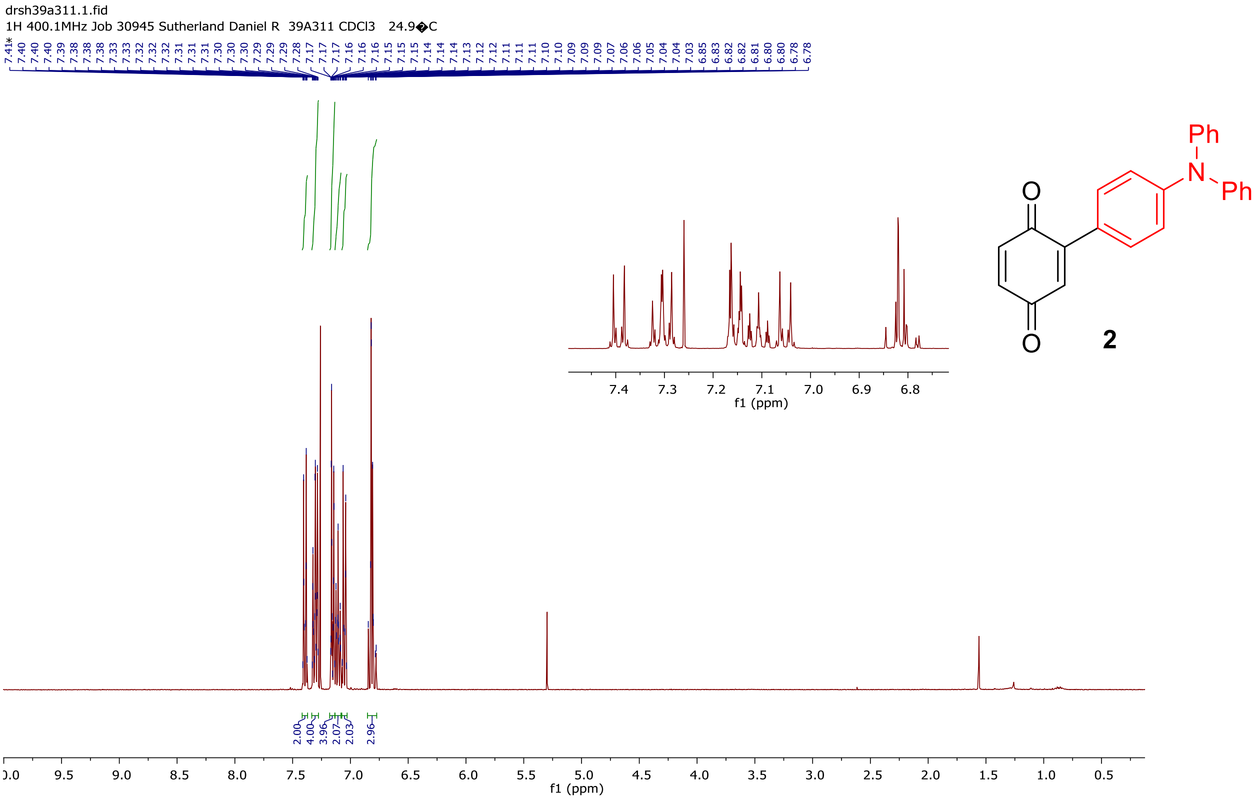

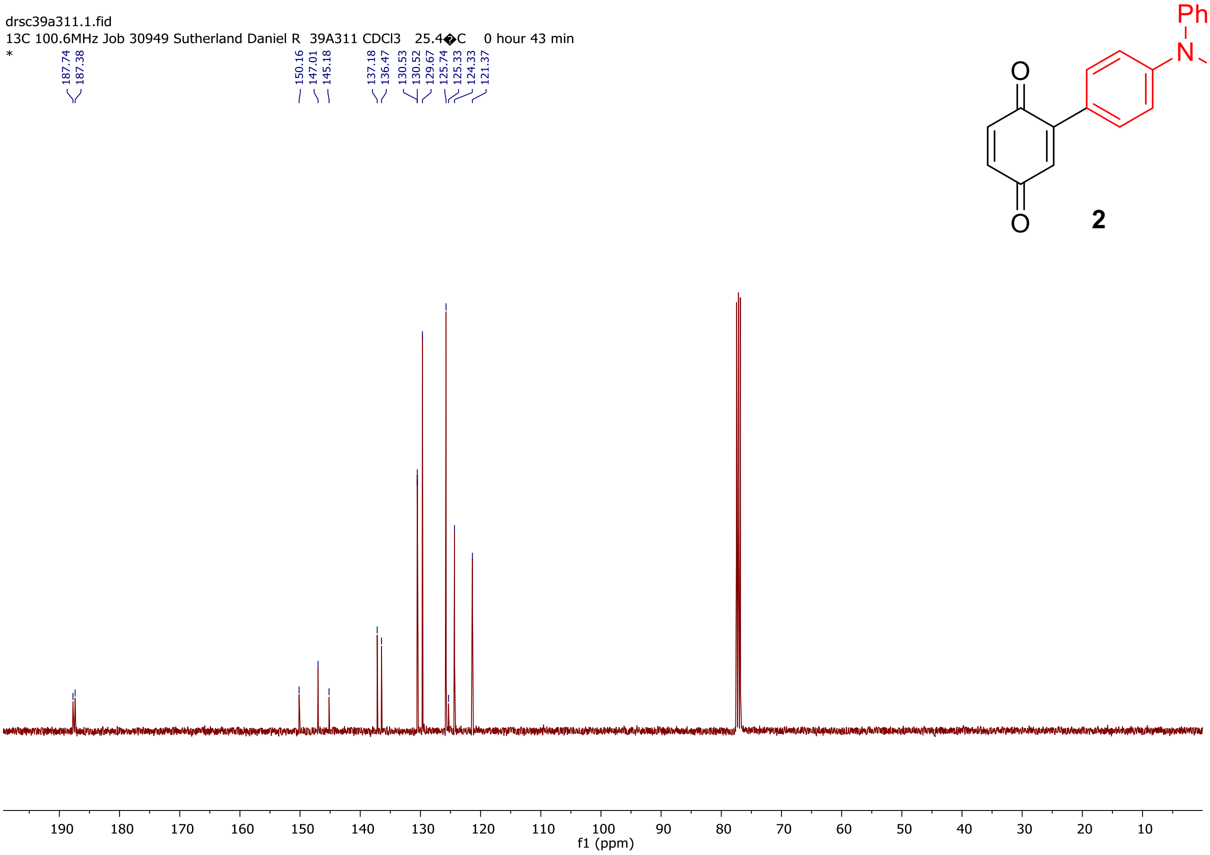

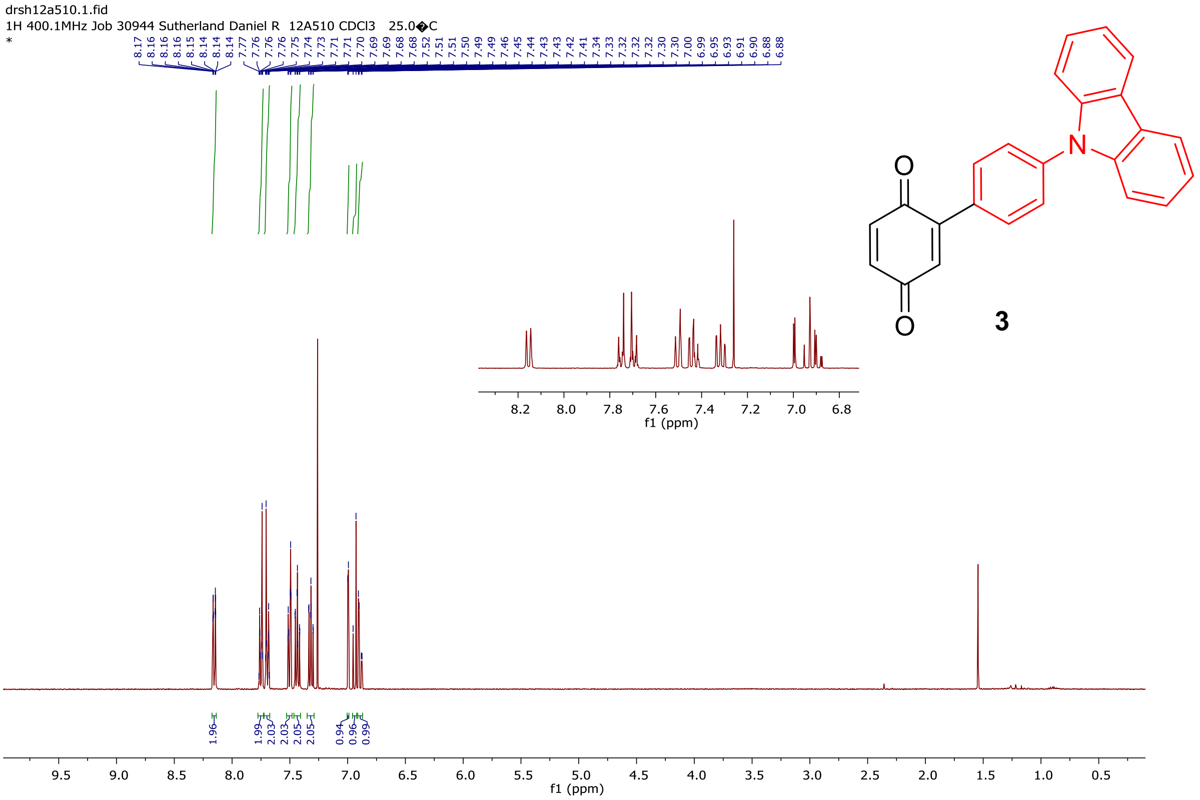
drsh12a510.1.fid
1H 400.1MHz Job 30944 Sutherland Daniel R 12A510 CDCl3 25.0�C
*
8.17 8.16 8.16 8.15 8.14 8.14 8.14 7.77 7.76 7.76 7.75 7.74 7.73 7.71 7.71 7.70 7.69 7.69 7.68 7.52 7.51 7.50 7.49 7.49 7.46 7.45 7.44 7.43 7.43 7.42 7.41 7.34 7.33 7.32 7.32 7.32 7.30 7.30 7.00 6.99 6.95 6.93 6.91 6.90 6.88 6.88
1.96 1.99 2.03 2.03 2.05 2.05 0.94 0.96 0.99
f1 (ppm)
O
N
3

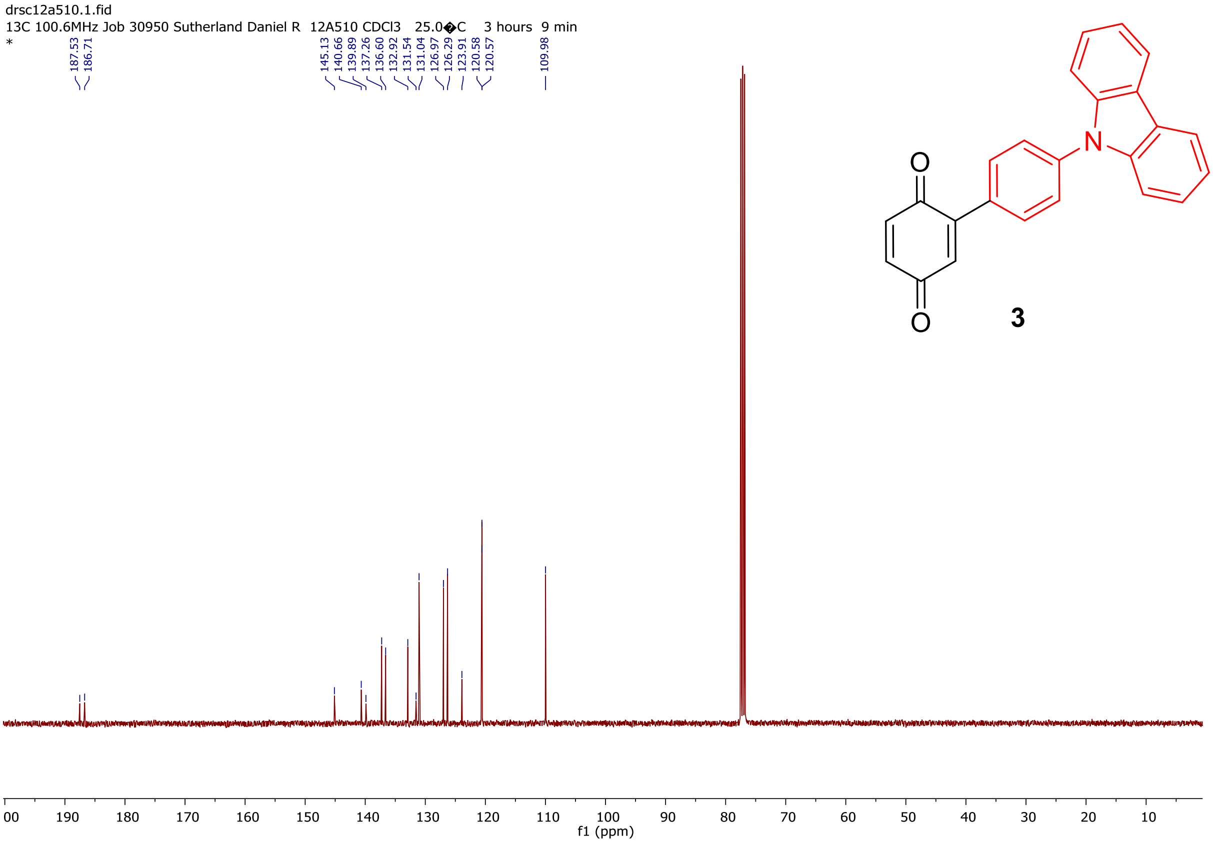
drsc12a510.1.fid
13C 100.6MHz Job 30950 Sutherland Daniel R 12A510 CDCl3 25.0�C 3 hours 9 min
*
187.53 186.71
145.13 140.66 139.89 137.26 136.60 132.92 131.54 131.04 126.97 126.29 123.91 120.58 120.57
109.98
f1 (ppm)
O
N
3

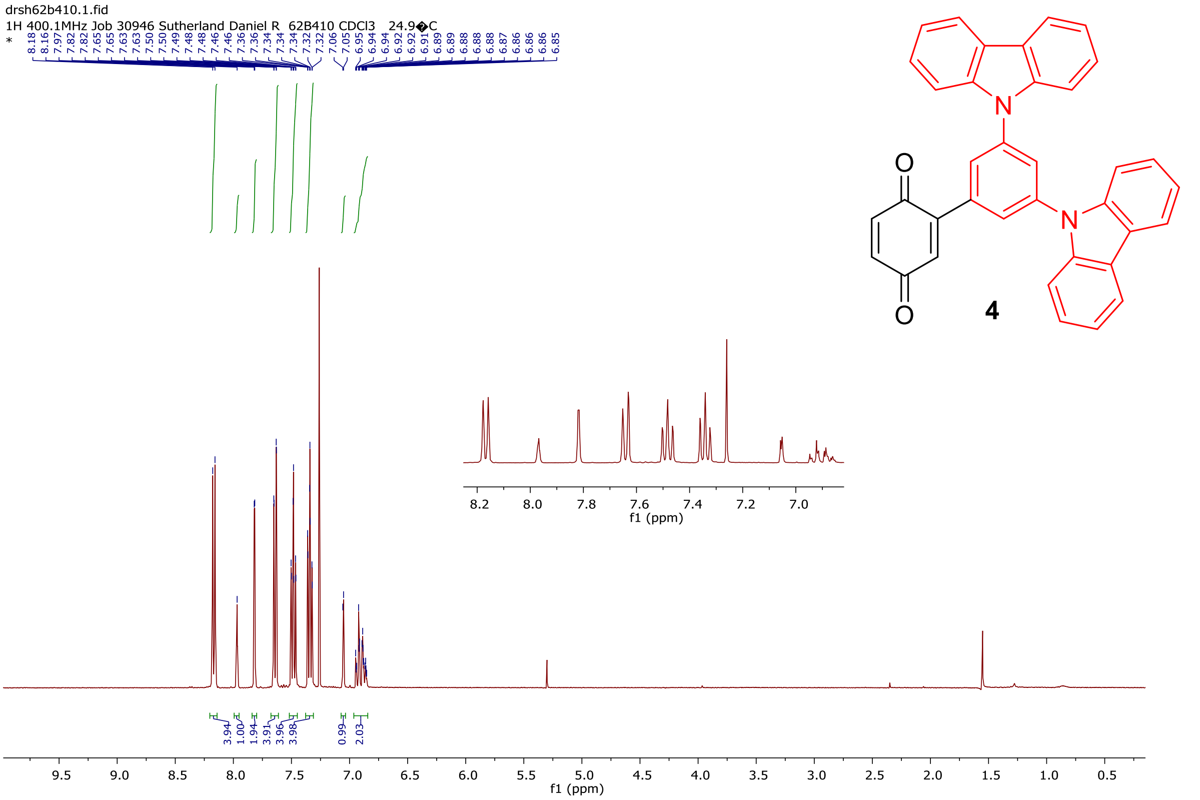
drsh62b410.1.fid
1H 400.1MHz Job 30946 Sutherland Daniel R 62B410 CDCl3 24.9�C
8.18 8.16 7.97 7.82 7.65 7.63 7.63 7.50 7.49 7.48 7.48 7.46 7.46 7.36 7.36 7.34 7.34 7.34 7.32 7.32 7.06 7.05 6.95 6.94 6.92 6.92 6.91 6.89 6.89 6.88 6.88 6.87 6.86 6.86 6.85
3.94 1.00 1.94 3.91 3.96 3.98 0.99 2.03
f1 (ppm)
O
N
4

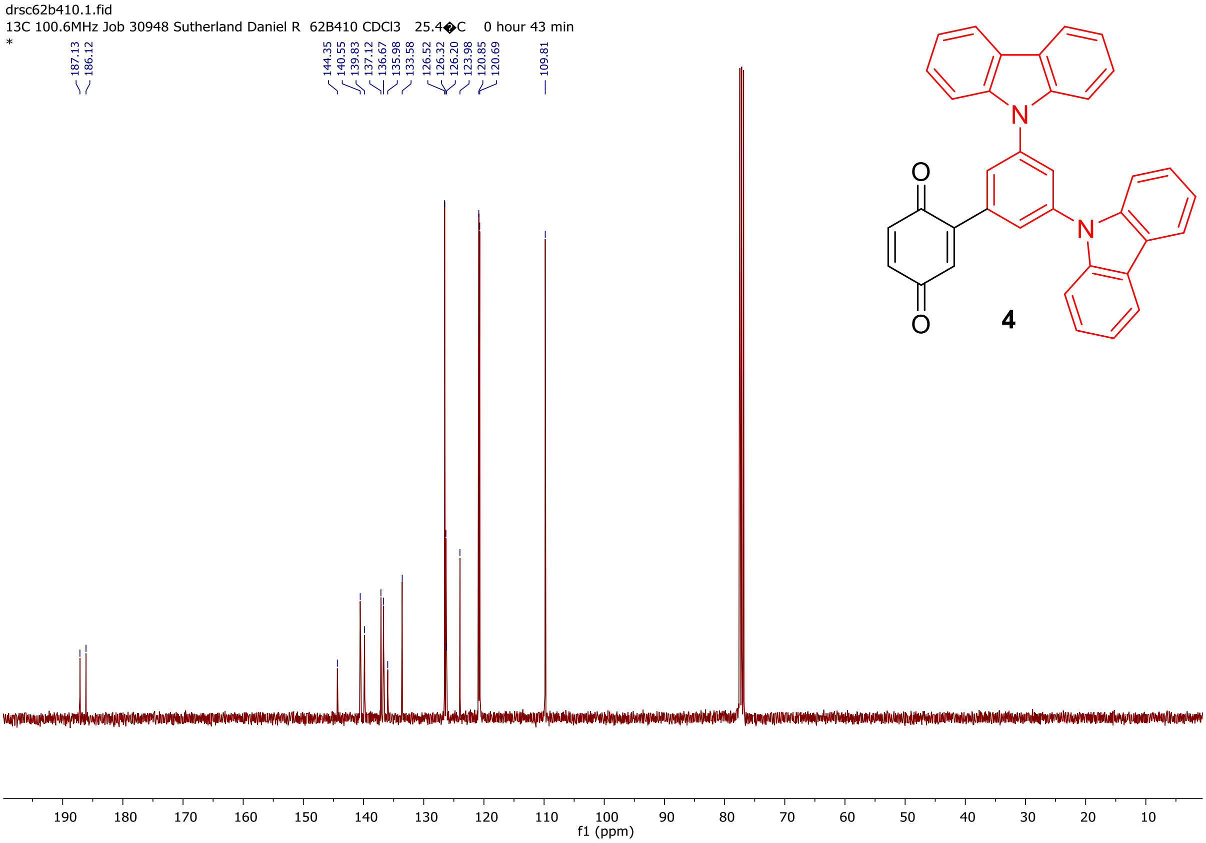
drsc62b410.1.fid
13C 100.6MHz Job 30948 Sutherland Daniel R 62B410 CDCl3 25.4�C 0 hour 43 min
187.13 186.12
144.35 140.55 139.83 137.12 136.67 135.98 133.58 126.52 126.32 126.20 123.98 120.85 120.69
109.81
f1 (ppm)
O
N
4

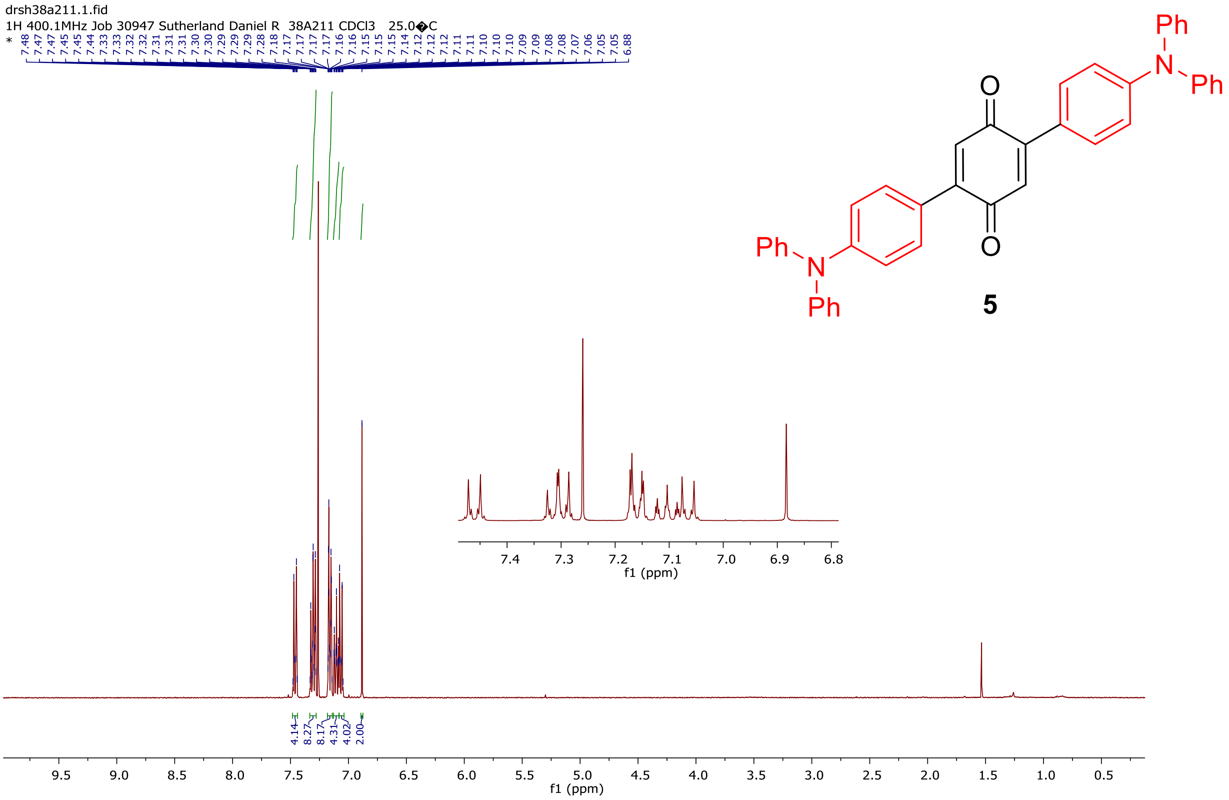
drsh38a211.1.fid
1H 400.1MHz Job 30947 Sutherland Daniel R 38A211 CDCl3 25.0�C
7.48 7.47 7.47 7.45 7.44 7.33 7.32 7.32 7.31 7.31 7.30 7.29 7.29 7.28 7.18 7.17 7.17 7.17 7.17 7.16 7.16 7.15 7.15 7.15 7.14 7.12 7.12 7.12 7.11 7.11 7.10 7.10 7.09 7.09 7.08 7.07 7.06 7.05 7.05 6.88
4.14 8.27 8.17 4.31 4.02 2.00
f1 (ppm)
Ph
N
Ph
O
O
Ph
N
Ph
5

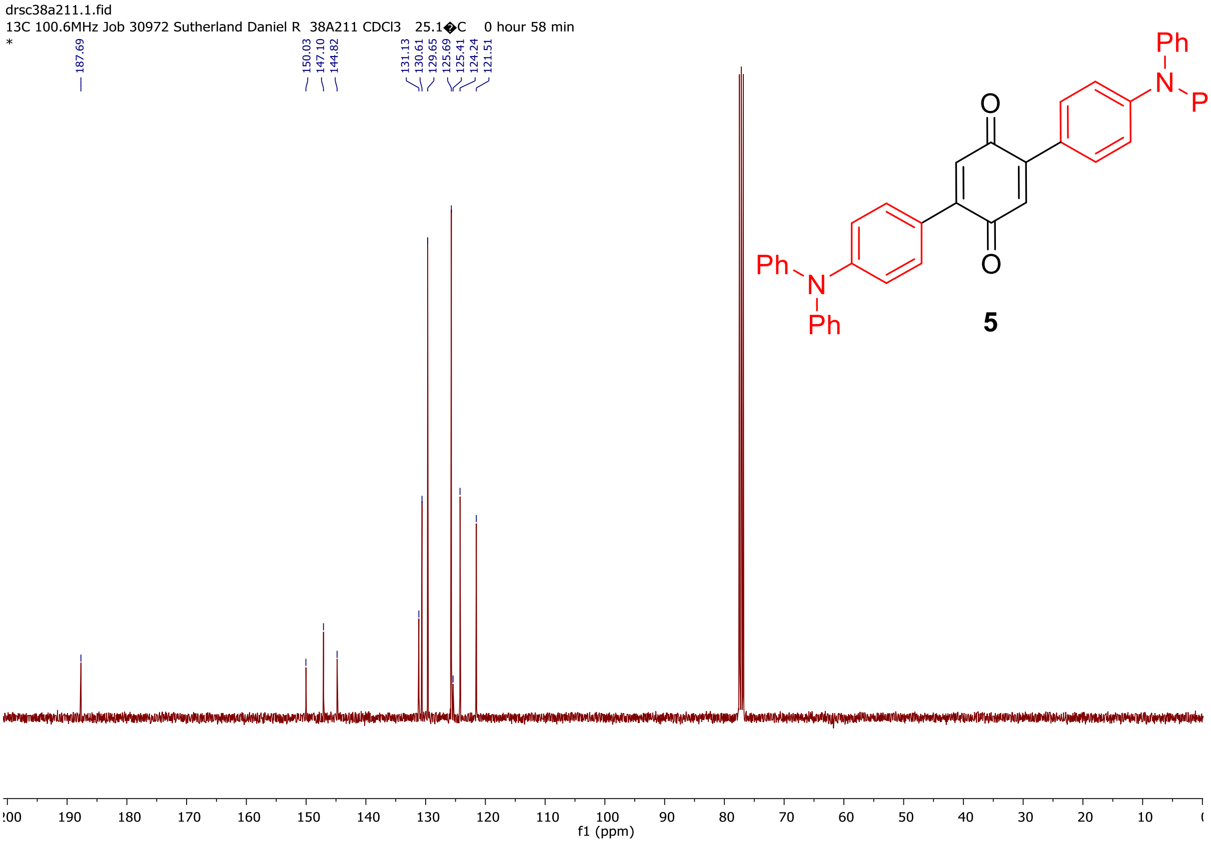
drsc38a211.1.fid
13C 100.6MHz Job 30972 Sutherland Daniel R 38A211 CDCl3 25.1�C 0 hour 58 min
187.69
150.03 147.10 144.82
131.13 130.61 129.65 125.69 125.41 124.24 121.51
f1 (ppm)
Ph
N
Ph
O
O
Ph
N
Ph
5

2. Photophysical characterisation

Photophysical measurements. Optically dilute solutions of concentrations in the order of 10^{-5} or 10^{-6} M were prepared in HPLC grade solvent for absorption and emission analysis. Absorption spectra were recorded at room temperature on a Shimadzu UV-1800 double beam spectrophotometer. Steady-state emission and time-resolved emission spectra were recorded at 298 K using an Edinburgh Instruments F980 fluorimeter. Samples were excited at 360 nm for steady-state measurements and at 378 nm for time-resolved measurements. Photoluminescence quantum yields for solutions were determined using the optically dilute method [4] in which four sample solutions with absorbance at 360 nm being ca. 0.10, 0.080, 0.060 and 0.040 were used. Their emission intensities were compared with those of a reference, quinine sulfate, whose quantum yield (Φ_r) in 1 *N* H_2SO_4 was determined to be 54.6% using absolute method [5]. The quantum yield of sample, Φ_{PL}, can be determined by the equation $\Phi_{PL} = \Phi_r(A_r/A_s)((I_s/I_r)(n_s/n_r)^2$, where A stands for the absorbance at the excitation wavelength (λ_{exc} = 360 nm), I is the integrated area under the corrected emission curve and n is the refractive index of the solvent with the subscripts "s" and "r" representing sample and reference respectively. An integrating sphere was employed for quantum yield measurements for thin film samples.

3. DFT modelling

Computational methodology

The calculations were performed with the Gaussian 09 [6] revision D.018 suite. Initially, the geometries of the compounds were fully optimised using DFT employing the PBE0 [7] functional with the Pople [8] 6-31G(d,p) basis set. Frequency calculations were further carried out to confirm the minima of all optimised geometries. No imaginary frequencies were found. Tamm–Dancoff excited-state calculations were then carried out using the same functional and basis set that was employed for the ground-state geometries in order to determine the excitation

energies of the first singlet (S_1) and triplet (T_1) excited states. The molecular orbitals were visualised using GaussView 5.0 software [9].

Table S1: Optimised atomic coordinates of **2** obtained from DFT calculations. Total Electronic Energy (EE) = -1128.68697719 a.u.

Center Number	Atomic Number	Atomic Type	Coordinates (Angstroms) X	Y	Z
1	6	0	-4.308226	-3.557286	-0.399363
2	6	0	-3.161635	-3.356689	-1.163917
3	6	0	-2.437495	-2.176331	-1.053680
4	6	0	-2.844979	-1.185895	-0.154052
5	6	0	-4.720949	-2.565195	0.486351
6	1	0	-4.875538	-4.477762	-0.494101
7	1	0	-2.834135	-4.118559	-1.865139
8	1	0	-1.552053	-2.012928	-1.659625
9	1	0	-5.610445	-2.711240	1.092009
10	6	0	-2.813901	1.241205	0.067693
11	6	0	-2.421165	2.205357	1.001638
12	6	0	-3.112300	3.407035	1.093209
13	6	0	-4.211622	3.654548	0.274897
14	6	0	-4.610652	2.688316	-0.645163
15	1	0	-1.573378	2.004323	1.648859
16	1	0	-2.797287	4.148405	1.821576

17	1	0	-4.753753	4.591476	0.354939
18	1	0	-5.463680	2.871252	-1.291875
19	7	0	-2.112619	0.017375	-0.034487
20	6	0	-0.712986	-0.000978	-0.006997
21	6	0	-0.019375	-1.071980	0.574953
22	6	0	0.026611	1.057975	-0.554631
23	6	0	1.363982	-1.086431	0.606399
24	1	0	-0.577625	-1.894141	1.009408
25	6	0	1.408635	1.035025	-0.519503
26	1	0	-0.492708	1.899331	-1.000271
27	6	0	2.112743	-0.036051	0.052233
28	1	0	1.873733	-1.918728	1.074312
29	1	0	1.959100	1.876089	-0.930527
30	6	0	3.578562	-0.045799	0.022106
31	6	0	4.280911	0.489649	-1.004942
32	6	0	4.341624	-0.632524	1.174299
33	6	0	5.746682	0.538046	-1.047434
34	1	0	3.781551	0.899472	-1.877964
35	6	0	5.820676	-0.607088	1.115327
36	6	0	6.481719	-0.056545	0.091284
37	1	0	6.320523	-1.055356	1.968938
38	1	0	7.565822	-0.019915	0.038005
39	8	0	3.787366	-1.107850	2.151894
40	8	0	6.345594	1.036979	-1.990374
41	6	0	-3.914361	1.491307	-0.757779

42	6	0	-3.992918	-1.389270	0.618072
43	1	0	-4.306468	-0.619345	1.315677
44	1	0	-4.216473	0.740586	-1.480965

Table S2: Optimised atomic coordinates of **3** obtained from DFT calculations. Total Electronic Energy (EE) = -1127.50609253 a.u.

Center Number	Atomic Number	Atomic Type	Coordinates (Angstroms) X	Y	Z
1	6	0	6.605485	-0.118799	-0.005262
2	6	0	5.873817	1.155589	-0.176420
3	6	0	4.404449	1.104318	-0.137011
4	6	0	3.704997	-0.041144	0.025745
5	6	0	4.463225	-1.324822	0.194351
6	6	0	5.941998	-1.266545	0.173576
7	1	0	7.689516	-0.059953	-0.030529
8	1	0	3.906233	2.065156	-0.228353
9	1	0	6.440328	-2.222476	0.304678
10	8	0	3.901395	-2.398428	0.334798
11	8	0	6.470482	2.210242	-0.335190
12	6	0	2.235937	-0.062969	0.014377
13	6	0	1.496619	-0.912270	0.850533
14	6	0	1.536154	0.808408	-0.832858
15	6	0	0.111274	-0.868754	0.857968

16	1	0	2.011941	-1.594714	1.514026
17	6	0	0.151624	0.838631	-0.847492
18	1	0	2.088821	1.439188	-1.522831
19	6	0	-0.572761	0.002262	0.006529
20	1	0	-0.451727	-1.498288	1.538905
21	1	0	-0.378719	1.485246	-1.538513
22	7	0	-1.976514	0.032115	0.001408
23	6	0	-2.765440	1.177816	0.087728
24	6	0	-2.813619	-1.078584	-0.094621
25	6	0	-2.381752	2.508369	0.242597
26	6	0	-4.126226	0.798389	0.048971
27	6	0	-2.485702	-2.424070	-0.248771
28	6	0	-4.156870	-0.640499	-0.069294
29	6	0	-3.388187	3.462218	0.329195
30	1	0	-1.336767	2.791598	0.304947
31	6	0	-5.117230	1.776221	0.139244
32	6	0	-3.531612	-3.333035	-0.347860
33	1	0	-1.453464	-2.752429	-0.301059
34	6	0	-5.188397	-1.574421	-0.171270
35	6	0	-4.742031	3.105686	0.272797
36	1	0	-3.115610	4.506537	0.447999
37	1	0	-6.166607	1.497570	0.110481
38	6	0	-4.869368	-2.918491	-0.303881
39	1	0	-3.303126	-4.387862	-0.466542
40	1	0	-6.225008	-1.250731	-0.152774

41	1	0	-5.502175	3.877382	0.341733
42	1	0	-5.661356	-3.656559	-0.382127

Table **S3**. Optimized atomic coordinates of **4** obtained from DFT calculations. Total Electronic Energy (EE) = -1643.23626398 a.u.

Center Number	Atomic Number	Atomic Type	Coordinates (Angstroms) X	Y	Z
1	6	0	-1.326232	5.560495	-0.585944
2	6	0	-1.199349	4.092816	-0.616442
3	6	0	-0.181454	3.430135	-0.028763
4	6	0	0.872138	4.215126	0.694752
5	6	0	0.754037	5.689573	0.701852
6	6	0	-0.268749	6.321790	0.114747
7	1	0	-1.977266	3.569244	-1.165244
8	1	0	1.547601	6.212463	1.227234
9	1	0	-0.375944	7.402360	0.119072
10	8	0	1.797551	3.675888	1.279314
11	8	0	-2.263250	6.127532	-1.125612
12	6	0	-0.092674	1.961003	-0.040604
13	6	0	1.134695	1.317817	-0.217814
14	6	0	-1.254252	1.200112	0.118702
15	6	0	1.201370	-0.074031	-0.211642
16	1	0	2.038693	1.892284	-0.368931
17	6	0	-1.187743	-0.191613	0.091025

18	1	0	-2.206707	1.683075	0.309123
19	6	0	0.039878	-0.833256	-0.063487
20	1	0	0.091674	-1.916089	-0.056324
21	7	0	2.444831	-0.710032	-0.350187
22	6	0	3.596227	-0.393545	0.374150
23	6	0	2.722329	-1.795585	-1.179367
24	6	0	3.793068	0.587911	1.344438
25	6	0	4.622548	-1.292204	0.006362
26	6	0	1.906959	-2.431306	-2.113948
27	6	0	4.067210	-2.183413	-0.984975
28	6	0	5.051577	0.664325	1.928661
29	1	0	3.006075	1.276072	1.633904
30	6	0	5.876385	-1.192423	0.610292
31	6	0	2.453485	-3.487246	-2.832838
32	1	0	0.885371	-2.111136	-2.287283
33	6	0	4.591608	-3.246335	-1.720786
34	6	0	6.085053	-0.210189	1.567928
35	1	0	5.233044	1.423652	2.683244
36	1	0	6.673117	-1.877789	0.336044
37	6	0	3.778535	-3.897886	-2.637562
38	1	0	1.837055	-4.000517	-3.564575
39	1	0	5.623640	-3.553987	-1.579632
40	1	0	7.055718	-0.118301	2.045048
41	1	0	4.172381	-4.728311	-3.214918
42	7	0	-2.360526	-0.950421	0.252534

43	6	0	-2.546362	-1.979286	1.173565
44	6	0	-3.534372	-0.787226	-0.478782
45	6	0	-1.672729	-2.468586	2.142079
46	6	0	-3.858036	-2.485640	1.030464
47	6	0	-3.809366	0.087360	-1.527919
48	6	0	-4.488128	-1.725712	-0.023881
49	6	0	-2.127843	-3.499484	2.954514
50	1	0	-0.677167	-2.056606	2.266686
51	6	0	-4.289599	-3.521445	1.859939
52	6	0	-5.072732	0.025228	-2.102479
53	1	0	-3.061903	0.784290	-1.892062
54	6	0	-5.749634	-1.767047	-0.618666
55	6	0	-3.418731	-4.026663	2.814992
56	1	0	-1.466266	-3.901256	3.715917
57	1	0	-5.294629	-3.921059	1.760604
58	6	0	-6.036933	-0.886552	-1.652268
59	1	0	-5.313987	0.697376	-2.920378
60	1	0	-6.493074	-2.482145	-0.278721
61	1	0	-3.740011	-4.833826	3.465620
62	1	0	-7.015072	-0.906072	-2.122229

Table S4: Optimised atomic coordinates of **5** obtained from DFT calculations. Total Electronic Energy (EE) = -1876.39063308 a.u.

Center	Atomic	Atomic	Coordinates (Angstroms)		
Number	Number	Type	X	Y	Z

1	6	0	-9.347732	-2.811117	-2.223097
2	6	0	-8.213788	-2.214819	-2.769063
3	6	0	-7.492313	-1.274765	-2.043927
4	6	0	-7.889603	-0.935390	-0.746569
5	6	0	-9.750223	-2.463284	-0.936226
6	1	0	-9.912996	-3.539516	-2.795867
7	1	0	-7.894305	-2.470760	-3.774970
8	1	0	-6.616602	-0.798886	-2.473527
9	1	0	-10.629594	-2.924998	-0.496985
10	6	0	-7.873531	0.994557	0.737826
11	6	0	-7.488946	1.315794	2.043577
12	6	0	-8.193346	2.275585	2.759855
13	6	0	-9.297690	2.909661	2.196070
14	6	0	-9.688117	2.579627	0.900748
15	1	0	-6.637217	0.809493	2.486594
16	1	0	-7.884584	2.517012	3.772670
17	1	0	-9.850042	3.653219	2.761948
18	1	0	-10.544733	3.070448	0.448275
19	7	0	-7.162650	0.020342	-0.000249
20	6	0	-5.762535	0.002961	0.007195
21	6	0	-5.059048	-1.205400	-0.098843
22	6	0	-5.031852	1.194603	0.127521
23	6	0	-3.675304	-1.223182	-0.092993
24	1	0	-5.609485	-2.136177	-0.183007

25	6	0	-3.649469	1.167371	0.134950
26	1	0	-5.557772	2.138425	0.221565
27	6	0	-2.934720	-0.034985	0.015265
28	1	0	-3.156432	-2.170253	-0.161961
29	1	0	-3.107284	2.100905	0.252429
30	6	0	-1.470198	-0.014962	-0.031249
31	6	0	-0.780060	1.016759	-0.568737
32	6	0	-0.677580	-1.150476	0.546495
33	6	0	0.682623	1.114385	-0.589584
34	1	0	-1.286441	1.856259	-1.035870
35	6	0	0.784801	-1.080303	0.470679
36	6	0	1.474796	-0.032211	-0.035171
37	1	0	1.291008	-1.960925	0.854853
38	8	0	-1.214020	-2.117151	1.071647
39	8	0	1.219562	2.113124	-1.050257
40	6	0	-8.978772	1.635183	0.169356
41	6	0	-9.024400	-1.538691	-0.195691
42	1	0	-9.329649	-1.274204	0.811642
43	1	0	-9.274356	1.383291	-0.844086
44	6	0	2.937950	0.015471	-0.030410
45	6	0	3.675708	0.645996	-1.045778
46	6	0	3.655296	-0.612532	1.000894
47	6	0	5.059165	0.624284	-1.042529
48	1	0	3.154277	1.140980	-1.854618
49	6	0	5.037018	-0.622373	1.021968

50	1	0	3.113434	-1.062278	1.827733
51	6	0	5.764772	-0.007166	-0.008333
52	1	0	5.608498	1.095869	-1.850247
53	1	0	5.567105	-1.090117	1.844582
54	7	0	7.164335	-0.016213	0.003558
55	6	0	7.868542	-1.115548	0.545557
56	6	0	7.462648	-2.424316	0.264811
57	6	0	8.987422	-0.902450	1.356933
58	6	0	8.159659	-3.499619	0.801413
59	1	0	6.600486	-2.587771	-0.373786
60	6	0	9.688794	-1.984780	1.873332
61	1	0	9.300299	0.114030	1.572836
62	6	0	9.277714	-3.287814	1.604209
63	1	0	7.834127	-4.510927	0.576225
64	1	0	10.556193	-1.806220	2.501952
65	1	0	9.824473	-4.130600	2.015197
66	6	0	7.894097	1.076462	-0.519070
67	6	0	7.498454	2.389402	-0.243965
68	6	0	9.028687	0.850470	-1.304512
69	6	0	8.222351	3.456680	-0.760565
70	1	0	6.622134	2.563136	0.372204
71	6	0	9.756656	1.924770	-1.800561
72	1	0	9.332861	-0.169496	-1.516539
73	6	0	9.356517	3.232254	-1.536916
74	1	0	7.903887	4.471437	-0.541128

75	1	0	10.636222	1.736478	-2.409124
76	1	0	9.923715	4.068915	-1.932444

4. References

1. O.V. Dolomanov, L. J. Bourhis, R. J. Gildea, J. A. K. Howard, and H. Puschmann, *J. Appl. Cryst.,* **2009**, *42,* 339-341.
2. G.M Sheldrick, *Acta Cryst*. **2008,** *A64,* 112-122.
3. G.M Sheldrick, , *Acta Cryst*. **2015,** *C71,* 3–8.
4. J. N. Demas and G. A. Crosby, *J. Phys. Chem.*, **1971,** *75,* 991-1024.
5. W. H. Melhuish, *J. Phys. Chem.,* **1961,** *65,* 229-235.
6. M. J. Frisch, G. W. Trucks, H. B. Schlegel, G. E. Scuseria, M. A. Robb, J. R. Cheeseman, G. Scalmani, V. Barone, B. Mennucci, G. A. Petersson, H. Nakatsuji, M. Caricato, X. Li, H. P. Hratchian, A. F. Izmaylov, J. Bloino, G. Zheng, J. L. Sonnenberg, M. Hada, M. Ehara, K. Toyota, R. Fukuda, J. Hasegawa, M. Ishida, T. Nakajima, Y. Honda, O. Kitao, H. Nakai, T. Vreven, J. A. Montgomery, Jr., J. E. Peralta, F. Ogliaro, M. Bearpark, J. J. Heyd, E. Brothers, K. N. Kudin, V. N. Staroverov, T. Keith, R. Kobayashi, J. Normand, K. Raghavachari, A. Rendell, J. C. Burant, S. S. Iyengar, J. Tomasi, M. Cossi, N. Rega, J. M. Millam, M. Klene, J. E. Knox, J. B. Cross, V. Bakken, C. Adamo, J. Jaramillo, R. Gomperts, R. E. Stratmann, O. Yazyev, A. J. Austin, R. Cammi, C. Pomelli, J. W. Ochterski, R. L. Martin, K. Morokuma, V. G. Zakrzewski, G. A. Voth, P. Salvador, J. J. Dannenberg, S. Dapprich, A. D. Daniels, O. Farkas, J. B. Foresman, J. V. Ortiz, J. Cioslowski, and D. J. Fox, Gaussian 09, Revision D.01. Wallingford, CT, 2013**.**
7. C. Adamo and V. Barone, *J. Chem. Phys*., **1999,** *110,* 6.
8. A. J. Pople, J. S. Binkley and R. Seeger, *Int. J. Quant. Chem. Symp*., **1976,** *10,* 1.
9. M. Moral, L. Muccioli, W. J. Son, Y. Olivier and J. C. Sancho-García, *J. Chem. Theory Comput*., **2015,** *11,* 168.
